# Multi-Elemental Analysis and Geographical Discrimination of Greek “Gigantes Elefantes” Beans Utilizing Inductively Coupled Plasma Mass Spectrometry and Machine Learning Models

**DOI:** 10.3390/foods13183015

**Published:** 2024-09-23

**Authors:** Eleni C. Mazarakioti, Anastasios Zotos, Vassilios S. Verykios, Efthymios Kokkotos, Anna-Akrivi Thomatou, Achilleas Kontogeorgos, Angelos Patakas, Athanasios Ladavos

**Affiliations:** 1 Department of Food Science and Technology, University of Patras, 30131 Agrinio, Greece; ekokkotos@upatras.gr (E.K.); athomatu@upatras.gr (A.-A.T.); apatakas@upatras.gr (A.P.); 2 Department of Sustainable Agriculture, University of Patras, 30131 Agrinio, Greece; azotos@upatras.gr; 3 School of Sciences and Technology, Hellenic Open University, 26335 Patras, Greece; verykios@eap.gr; 4 Department of Agriculture, International Hellenic University, 57001 Thessaloniki, Greece; akontoge@ihu.gr

**Keywords:** elemental fingerprint, giant beans, multi-elemental analysis, ICP-MS, geographical origin, machine learning, decision tree

## Abstract

Greek giant beans, also known as “Gigantes Elefantes” (elephant beans, *Phaseolus vulgaris* L.,) are a traditional and highly cherished culinary delight in Greek cuisine, contributing significantly to the economic prosperity of local producers. However, the issue of food fraud associated with these products poses substantial risks to both consumer safety and economic stability. In the present study, multi-elemental analysis combined with decision tree learning algorithms were investigated for their potential to determine the multi-elemental profile and discriminate the origin of beans collected from the two geographical areas. Ensuring the authenticity of agricultural products is increasingly crucial in the global food industry, particularly in the fight against food fraud, which poses significant risks to consumer safety and economic stability. To ascertain this, an extensive multi-elemental analysis (Ag, Al, As, B, Ba, Be, Ca, Cd, Co, Cr, Cs, Cu, Fe, Ga, Ge, K, Li, Mg, Mn, Mo, Na, Nb, Ni, P, Pb, Rb, Re, Se, Sr, Ta, Ti, Tl, U, V, W, Zn, and Zr) was performed using Inductively Coupled Plasma Mass Spectrometry (ICP-MS). Bean samples originating from Kastoria and Prespes (products with Protected Geographical Indication (PGI) status) were studied, focusing on the determination of elemental profiles or fingerprints, which are directly related to the geographical origin of the growing area. In this study, we employed a decision tree algorithm to classify Greek “Gigantes Elefantes” beans based on their multi-elemental composition, achieving high performance metrics, including an accuracy of 92.86%, sensitivity of 87.50%, and specificity of 96.88%. These results demonstrate the model’s effectiveness in accurately distinguishing beans from different geographical regions based on their elemental profiles. The trained model accomplished the discrimination of Greek “Gigantes Elefantes” beans from Kastoria and Prespes, with remarkable accuracy, based on their multi-elemental composition.

## 1. Introduction

Greek giant beans (*Phaseolus vulgaris* L.), known as “Gigantes Elefantes” or elephant beans, are a unique variety of legume that holds a prominent place in the Greek diet. These large, creamy-white beans are grown primarily in the regions of Kastoria and Prespes, in Western Macedonia, Greece, where the climate and soil conditions contribute to their distinct flavor and texture as well as to their special organoleptic characteristics. Visually, the beans have a substantial size, measuring approximately 2 to 3 cm in length and possessing a round, oblong shape. They are an excellent source of plant-based protein, dietary fiber, and essential minerals such as Iron, Magnesium, and Potassium. Furthermore, they possess low fat content and contain no cholesterol, making them a healthy and versatile ingredient for various dietary preferences, including vegan and vegetarian lifestyles [1,2]. Beans from both geographical areas have received the Protected Geographical Indication (PGI) status with the following registered names and specifications: (1) Fasolia Gigantes Elefantes Prespon Florinas {112/18-01-1994 (O.J. no. 25/18-01-94), 301066/13-05-2009 (O.J. no. 1007 Β)}, and (2) Fasolia Gigantes Elefantes Kastorias {L203/2003} [3]. Gigantes Elefantes are also exported to other countries, allowing individuals worldwide to savor the unique flavors and textures these beans offer.

The cultivation of Gigantes Elefantes beans takes place at small-scale farms, where in several cases traditional techniques are used. The areas of Kastoria and Prespes provide an ideal environment for bean cultivation. Kastoria is located on the western shore of Lake Orestiada, which has a significant impact on the local climate and soil conditions [4]. The Prespes region, located in the northwestern part of Greece, near the borders with Albania and North Macedonia, is known for its rich biodiversity, including numerous species of birds and fish. It is renowned for its two large lakes, Great Prespa and Lesser Prespa. Generally, both areas are characterized by a continental microclimate, with hot/dry summers and cold/wet winters; however, the moderating effect of the lakes in each region does not allow extreme temperature changes [5]. Due to their intriguing geomorphological diversity, the soil conditions in Kastoria and Prespes vary depending on the specific location, but they generally consist of the following types: alluvial, clay, loam, and mountain soil [4]. These particular soil types provide optimal conditions for cultivating beans with unique characteristics, hence the production of PGE products. These distinct characteristics are also reflected at the chemical level, as it is well established that the elemental composition of agricultural products can vary significantly depending on the specific geographical region in which they are cultivated [6,7,8]. The elements are absorbed through the roots from the soil and transferred to the other parts of the plant [9,10]. The final composition of the absorbed elements mainly reflects their availability in the soil, leading to distinctive elemental patterns or signatures associated with specific geographic areas. Therefore, the elemental profile can be used to predict the geographical origin of an agricultural product [11]. 

In recent years, the issue of food fraud has gained global attention, as fraudulent practices threaten the safety, authenticity, and economic value of agricultural products [12]. As a result, there is an urgent need for reliable methods to authenticate the geographical origin of these beans and protect their PGI status. This study addresses this need by employing multi-elemental analysis and machine learning models to develop a robust elemental fingerprinting technique, offering a novel approach to food authenticity and safety. This can be a very useful tool since fraudulent practices have been reported [13], in the form of substituting authentic beans with lower-grade varieties or mixing them with other types of beans, with a direct impact on human health and economic losses.

The PGI “Gigantes Elefantes” beans are crucial from an economic point of view for the producers of Kastoria and Prespes as the products are more expensive than other common beans that are mainly imported, making them vulnerable to mislabeling and adulteration practices. It is more than obvious that the development of trustworthy elemental techniques for food analysis to guarantee the origin and authenticity, and thus the quality, of agricultural products is required. Lately, Inductively Coupled Plasma Mass Spectroscopy (ICP-MS) has been utilized for the determination of the elemental composition of a huge variety of agricultural and food products (such as wine [6,14], rice [15,16], and olive oil [17]) and thus, it is considered a valuable analytical technique for geographical origin authentication [11]. It is gaining widespread acceptance in food forensics due to its high sensitivity and accuracy in combination with the low detection limits. Although several studies have been carried out on multi-elemental analysis of various legumes (i.e., fava Santorinis [18,19], common beans [20,21], etc.) using ICP-MS, to the best of our knowledge, there are not any publications that report the elemental composition of the PGI “Gigantes Elefantes” of Kastoria and Prespes for geographical origin discrimination reasons.

Recent advancements in supervised learning and classification techniques have shown significant potential in precision agriculture, particularly in ensuring the authenticity and quality of agricultural products [22,23]. Among these techniques, decision tree learning algorithms have gained popularity due to their simplicity and interpretability. Decision trees are considered white-box models, meaning their decision-making process can be easily understood and interpreted by domain experts. This is particularly important in agriculture, where expert knowledge is crucial for validating model outputs. In our study, a simple decision tree algorithm is employed to classify and predict the geographical origin of PGI “Gigantes Elefantes” beans based on their elemental profiles. Decision trees provide a clear visualization of the decision rules and the importance of different elements in determining the origin, making the results accessible and actionable for stakeholders in the agricultural sector. This approach aligns with recent studies that highlight the efficacy of decision trees in agricultural applications, such as crop classification and soil analysis [24,25].

The development of reliable analytical techniques for food analysis is crucial to guarantee the origin and authenticity of agricultural products, which in turn ensures their quality. In this context, multi-elemental analysis combined with advanced machine learning models offers a powerful approach for creating chemical fingerprints that can verify the geographical origin of crops. By employing Inductively Coupled Plasma Mass Spectrometry (ICP-MS) to perform comprehensive elemental analyses, followed by the application of machine learning algorithms to classify the data, researchers can accurately determine the provenance of agricultural products. This methodology not only supports the fight against food fraud but also promotes fair trade practices and consumer trust. Ensuring the authenticity of PGI “Gigantes Elefantes” beans from Kastoria and Prespes not only protects the economic interests of local producers but also preserves the cultural heritage and culinary traditions associated with these unique beans.

The aim of this study is to determine the elemental profiles of PGI “Gigantes Elefantes” beans of Kastoria and Prespes and investigate the ability of ICP-MS analysis combined with decision trees to discriminate beans produced in the two areas of Greece. Establishing a chemical fingerprint, which will serve as a reference profile, offers a reliable method for certifying the geographical authenticity of PGI “Gigantes Elefantes”, aiding in the protection of their designation of origin and supporting fair trade practices. For this purpose, ICP-MS analysis is utilized providing accurate results in determining the concentration of various elements present in the samples. An extensive multi-elemental analysis which included 37 elements in total (Ag, Al, As, B, Ba, Be, Ca, Cd, Co, Cr, Cs, Cu, Fe, Ga, Ge, K, Li, Mg, Mn, Mo, Na, Nb, Ni, P, Pb, Rb, Re, Se, Sr, Ta, Ti, Tl, U, V, W, Zn, and Zr) was performed. The resulting data were analyzed using decision tree algorithms.

## 2. Materials and Methods

### 2.1. Sampling and Harvesting Area

In total, 120 and 160 samples of “Gigantes Elefantes” were harvested from the regions of Kastoria and Prespes, respectively, during September 2021 and 2022. The average value of the two harvesting years for each analyte was included in the analysis. Beans from Kastoria and Prespes were randomly collected in the field by our team. Each sample consisted of approximately 1 kg of giant beans. The producers in both regions followed traditional practices for cultivation. The collected samples were frozen at −18 °C for 7 days to induce chill shock, in order to remove any biotic factors that could potentially destroy our samples.

**Figure 1:**
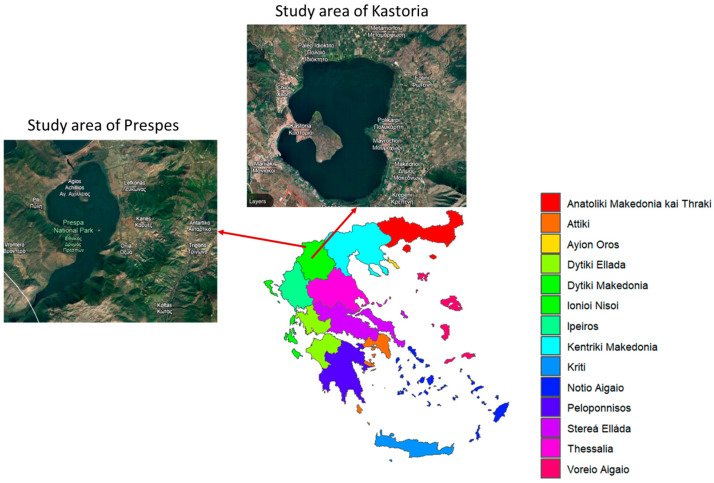
The selected geographical areas where PGI “Gigantes Elefantes” of Kastoria and Prespes are cultivated. [Pictures of study areas: Google Earth].

The Regional Unit of Kastoria in Greece has become a prominent region for the Protected Geographical Indication of pulse crops, specifically for dry giant beans. It is located in the northwestern part of Greece (Figure 1). Due to the captivating geomorphological diversity of the area, it was considered an excellent candidate for our research. The altitude, slope of the farmland, and distance from the lake exhibit significant variations across the area. Consequently, the microclimate and soil texture are anticipated to differ among subregions, thereby exerting a notable influence on the local production of beans, providing them with special organoleptic characteristics.

Similarly, the Regional Unit of Prespes is located in northern Greece near the borders with Albania and North Macedonia (Figure 1), and it is renowned for its agricultural productivity due to its unique geomorphological diversity and favorable microclimate. The area is situated between two lakes, the Lesser and Great Prespa lakes, surrounded by mountains and fertile plains. More than 50% of Greece’s biodiversity is concentrated in the ecosystem of the Prespa lakes. These geomorphological conditions develop the ideal microclimatic conditions, in conjunction with sufficient water supply from the lakes, to produce the optimal environment for bean cultivation.

### 2.2. Instrumentation

Quantitative elemental analyses of 37 analytes (Ag, Al, As, B, Ba, Be, Ca, Cd, Co, Cr, Cs, Cu, Fe, Ga, Ge, K, Li, Mg, Mn, Mo, Na, Nb, Ni, P, Pb, Rb, Re, Se, Sr, Ta, Ti, Tl, U, V, W, Zn, and Zr) were performed with a quadrupole ICP-MS Agilent Technologies 7850 series model (Santa Clara, CA, USA). Simultaneously, automated semiquantitative analyses (IntelliQuant Method) were performed for the majority of elements throughout the periodic table (78 elements). However, only the concentrations of the ones which were presented on the calibration standard solutions were calculated [26]. In the case of Lead, all three isotopes of Pb (^206^Pb, ^207^Pb, and ^208^Pb) were monitored for possible interferences to be reduced. ICP-MS operates at 1550 W RF power, 8.0 mm sampling depth, 1.05 L/min nebulizer gas, 4.3 mL/min helium cell gas, 3.0 V energy discrimination, and 2 °C S/C temperature (Table S1, available in Supplementary Materials). For data extraction, MassHunter 5.1 software (Agilent Technologies) was used.

The pulverization of the samples was performed by a laboratory mill (Pulverisette 11, Fritsch GmbH Milling and Sizing, Idar-Oberstein, Germany).

Sample digestions were carried out using the microwave oven Multiwave Go Plus, Anton Paar GmbH, Graz, Austria.

The Smart2Pure Water Purification System (Thermo Electron LED GmbH, Langenselbold, Germany) was utilized to obtain ultra-pure water suitable for trace element analysis.

### 2.3. Reagents, Standards, and Quality Assurance

Highly pure nitric acid (70% *v*/*v* TraceSELECT, for trace analysis) was purchased from Honeywell Fluka (Seelz, Germany); multi-elemental calibration standard 2A (100 mL: 10 µg/mL of Ag, Al, As, Ba, Be, Ca, Cd, Co, Cr, Cs, Cu, Fe, Ga, K, Li, Mg, Mn, Na, Ni, Pb, Rb, Se, Sr, Tl, U, V, Zn in a matrix of 5% HNO_3_), multi-elemental calibration standard 4 (10 µg/mL B, Ge, Mo, Nb, P, Re, S, Si, Ta, Ti, W, Zr in HNO_3_/trace HF), Rh ICP-internal standard solution (100 mL: 10 µg/mL in 2% HCl), and tuning solution (500 mL: 10 µg/mL Li, Y, Ce, Tl, and Co HNO_3_) were purchased from Agilent (Santa Clara, CA, USA). The multi-elemental stock standards 2A and 4 were used to prepare mixed calibration standard solutions of concentrations 2, 10, 50, 100, and 600 µg/L for a five-point calibration curve to be obtained. The latter solutions were diluted with 1% *v*/*v* HNO_3_ in ultra-pure 18.2 MΩ cm water. The limits of detection (LODs) and limits of quantification (LOQs) for all the analytes are presented in Table S2, and their values were determined by analyzing a series of ten blank samples. LOD and LOQ values were determined by multiplying the standard deviation of blank measurements by 3.3 and 10, respectively [27]. These blank samples were prepared using the same preparation procedure. The reference material IRMM-804 Rice Flour (European Commission, Joint Research Centre, Institute for Materials and Measurements, Geel, Belgium) was utilized to evaluate the accuracy of the method. It is prepared according to the sample experimental procedure. At least one sample of reference material was analyzed after a batch (approx. 10 samples). The obtained recovery data (Table S3) of the selected metals (based on reference material) were within the accepted values (80–120% of the certified values).

### 2.4. Sample Preparation and Microwave Digestion

**Figure 2:**
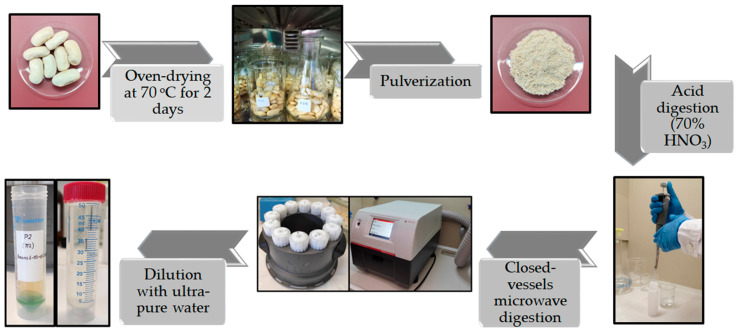
Sample preparation procedure.

Each of the samples was dried in an oven at 70 °C for 2 days to reduce the contained moisture. After drying, all the bean samples were pulverized, yielding a fine powder. A total of 0.5 g of each bean powder was transferred into Teflon vessels and digested with 4 mL of pure HNO_3_ (70%) in a microwave oven under the following conditions: the temperature was steadily increased for 15 min until it reached 180 °C, then the temperature was kept stable at 180 °C for 20 min following the cooling of the samples until they reached room temperature. The resulting digested samples were greenish and clear solutions, which were transferred into 50 mL polypropylene vials and further diluted to 50 mL with 18.2 MΩ·cm water. The final solutions were stored in the dark at 4 °C until analysis. All the reusable containers, during the experimental procedure, were acid-washed with diluted HNO_3_ in ultra-pure water prior to their use to avoid contamination. In Figure 2, a schematic representation of the whole experimental procedure of sample preparation is depicted.

### 2.5. Machine Learning Analysis Process

In this section, we detail the steps involved in our machine learning analysis process. This includes a comprehensive description of the dataset, the data preprocessing techniques applied, and the model training procedures. The goal is to accurately classify PGI “Gigantes Elefantes” beans based on their multi-elemental composition to determine their geographical origin and harvest year.

#### 2.5.1. Dataset Description

The dataset used in this study consists of multi-elemental analysis results of PGI “Gigantes Elefantes” beans collected from the regions of Kastoria and Prespes in Greece. The dataset includes measurements for 37 different elements (e.g., Ag, Al, As, B, Ba, Be, Ca, Cd, Co, Cr, Cs, Cu, Fe, Ga, Ge, K, Li, Mg, Mn, Mo, Na, Nb, Ni, P, Pb, Rb, Re, Se, Sr, Ta, Ti, Tl, U, V, W, Zn, and Zr) obtained using Inductively Coupled Plasma Mass Spectrometry (ICP-MS). Each sample is also associated with its geographical region of origin and the harvest year (2021 or 2022). This dataset provides the basis for training and testing the machine learning models.

#### 2.5.2. Data Preprocessing and Model Training

Before applying any machine learning models, the data underwent preprocessing to ensure accuracy and consistency. This involved handling missing values by removing incomplete records, as well as normalizing the data to bring all features to a common scale, which is essential for the convergence of some machine learning algorithms. The ‘Region’, ‘Villages’, and ‘Α/Α sample’ columns were excluded from the features used in the model, as they are identifiers rather than predictive features. Feature selection was then performed to identify the most informative elements for geographical discrimination.

#### 2.5.3. Model Selection and Training

A decision tree algorithm was selected for this study due to its simplicity, interpretability, and effectiveness in handling categorical data. Decision trees work by recursively partitioning the data into subsets based on the value of the most significant feature at each node. This process continues until the model can no longer partition the data or a stopping criterion is met (e.g., a maximum depth of the tree or a minimum number of samples per node).

In this study, the decision tree model was trained on 70% of the data, with the remaining 30% used for testing. The training process involved using the Gini impurity criterion to select the best splits at each node. Gini impurity measures the likelihood of an incorrect classification of a randomly chosen element if it were randomly labeled according to the distribution of labels in the dataset. The tree was pruned to prevent overfitting by setting a maximum depth and minimum samples per leaf node. Hyperparameters such as the maximum depth of the tree and the minimum number of samples per node were optimized using grid search in conjunction with cross-validation on the training data.

#### 2.5.4. Model Evaluation

After training the model, it was evaluated on the test set using several metrics: accuracy, sensitivity (also known as recall), and specificity. Accuracy measures the proportion of correct predictions out of all predictions made, sensitivity evaluates the model’s ability to correctly identify positive cases (beans from Prespes, for example), and specificity measures the model’s ability to correctly identify negative cases (beans from Kastoria). The confusion matrix provides a detailed breakdown of the model’s performance across these metrics.

#### 2.5.5. Feature Importance Analysis

Feature importance analysis was conducted to determine which elements most significantly influenced the model’s decisions. This was achieved by examining the frequency and impact of each feature (element) in the decision tree’s splits. Nickel (Ni), Manganese (Mn), and Rubidium (Rb) emerged as the most influential elements in distinguishing between beans from Kastoria and Prespes, suggesting that these elements are key indicators of geographical origin.

## 3. Results and Discussion

### 3.1. Multi-Elemental Analysis

The resulting data of the elemental composition of “Gigantes Elefantes” beans from Kastoria and Prespes, for the years 2021 and 2022, are reported in Tables S4 and S5, respectively. For each study area and harvesting year, the minimum, maximum, and mean values and the standard deviations are listed. Notably, the vast majority of the concentrations of elements in each area follow a similar trend during each harvesting year (Figures S1–S4). This fact allowed us to work with the average elemental concentrations of the two harvesting years for each study region.

The data analysis of the samples from Prespes and Kastoria revealed distinct differences and some similarities in the measured elemental concentrations. Generally, Potassium (K) is the most abundant macro-element in both areas, notably higher in Prespes with an average value of 38,116.19 mg/kg compared to 37,186.45 mg/kg in Kastoria. Similarly, Phosphorus (P) is higher in Prespes at 10,527.75 mg/kg than in Kastoria, 9090.27 mg/kg, followed by Magnesium (Mg) with average values of 3619.44 mg/kg in Kastoria and 3591.48 mg/kg in Prespes, and Calcium (Ca) with average values of 553.41 mg/kg in Prespes and 472.02 mg/kg in Kastoria. Iron (Fe) shows a higher concentration of 93.25 mg/kg in Prespes compared to 86.41 mg/kg in Kastoria.

Certain elements exhibit higher concentrations in Kastoria: Zinc (Zn) at 48.40 mg/kg in Kastoria compared to Prespes with a value of 39.48 mg/kg; Copper (Cu) and Strontium (Sr) are more concentrated in Kastoria, with values of 11.52 mg/kg and 8.39 mg/kg, respectively, against 9.51 mg/kg and 5.64 mg/kg in Prespes. Likewise, Molybdenum (Mo), Nickel (Ni), and Silver (Ag) show higher averages in the region of Kastoria. Molybdenum (Mo) is significantly higher in Kastoria (5.66 mg/kg) compared to Prespes (2.39 mg/kg). Nickel (Ni) and Silver (Ag) are also more abundant in Kastoria at 3.53 mg/kg and 0.009 mg/kg, respectively, compared to 2.20 mg/kg and 0.006 mg/kg in Prespes. The remaining elements were either present in much lower concentrations or were below the quantification limit (they are noted with 0).

According to our knowledge, there are currently no reports studying the multi-elemental profile of giant beans. Given the lack of literature on the elemental composition of the “Gigantes Elefantes” beans, we make an attempt to compare our findings with those of white common beans, a species within the same genus. Both studies we included utilized samples collected from Slovenia [20,28] (Table 1). Generally, based on the available literature, elemental compositions are consistent with literature data. However, Magnesium (Mg), Potassium (K), and Calcium (Ca) levels in both Kastoria and Prespes beans are higher than those reported in the literature for white common beans, indicating a potential regional enrichment in these elements. Iron (Fe) and Zinc (Zn) concentrations in both regions are also higher, suggesting that beans from these areas are richer in these essential nutrients. This variation in elemental composition could be attributed to variations in soil composition, environmental conditions, and cultivation practices between the two regions.

### 3.2. Decision Tree Model Training

**Figure 3:**
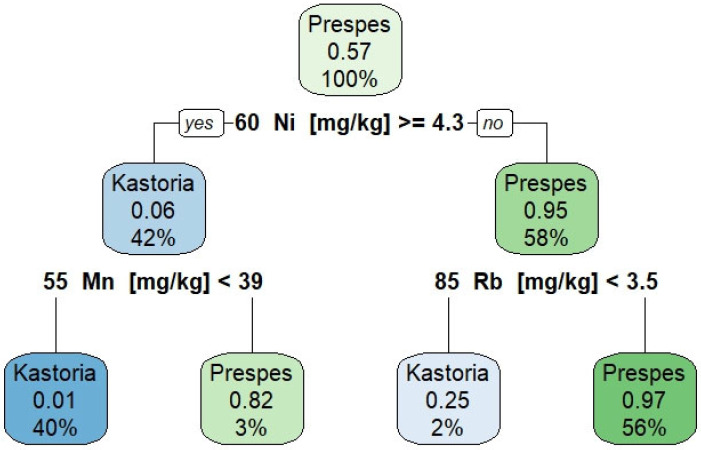
The decision tree model illustrates the hierarchical structure of decisions made to predict the geographical region of Greek “Gigantes Elefantes” beans based on their multi-elemental composition.

A decision tree model was trained on the training dataset (70% of the data) and tested on the remaining 30%. The model’s simplicity and interpretability make it suitable for domain experts. The decision tree model (Figure 3) classifies “Gigantes Elefantes” beans from Kastoria and Prespes based on concentrations of Nickel (Ni), Manganese (Mn), and Rubidium (Rb). The root node splits on Ni concentration, with further splits on Mn and Rb concentrations. For nodes labeled as Kastoria, the decimal number in the middle represents the percentage of Prespes samples, and vice versa. The tree indicates that higher Ni concentrations and specific Mn and Rb thresholds significantly distinguish the regions. The results show that beans with Ni ≥ 4.3 mg/kg and Mn < 39 mg/kg are likely from Kastoria, whereas those with Ni ≥ 4.3 mg/kg and Mn ≥ 39 mg/kg, or Ni < 4.3 mg/kg and Rb ≥ 3.5 mg/kg, are predominantly from Prespes. Beans with Ni < 4.3 mg/kg and Rb < 3.5 mg/kg are likely from Kastoria.

### 3.3. Model Prediction and Evaluation

The trained model was used to predict the ‘Region’ for the test dataset. The predictions were evaluated using a confusion matrix to determine the model’s accuracy, sensitivity, and specificity. The confusion matrix is shown below (Table 2). The confusion matrix and associated statistics indicate that the decision tree model performs well in distinguishing between beans from Kastoria and Prespes. The model achieves high accuracy (92.86%), sensitivity (87.50%), and specificity (96.88%).

**Figure 4:**
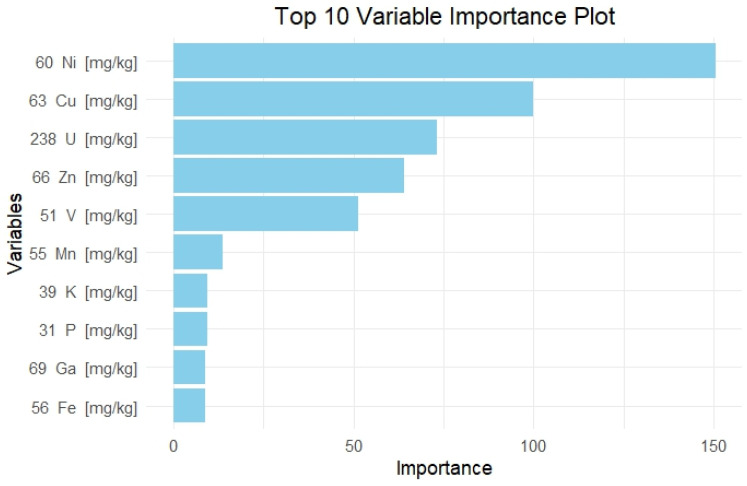
Feature importance graph using the decision tree model highlights the significance of each elemental feature in the classification process.

In order to shed more light on the findings of our process, we present a feature importance graph, which is an essential tool in understanding the behavior and decision-making process of machine learning models, especially decision trees. The variable importance graph, which is shown below (Figure 4), provides a visual representation of the most influential features, allowing us to identify which variables the model relies on most for making predictions. In the context of classifying “Gigantes Elefantes” beans into regions (Kastoria and Prespes), the graph highlights the top 10 most important features, with Nickel (Ni) being the most significant, followed by Copper (Cu), Uranium (U), and Zinc (Zn). This insight is crucial for feature selection, model refinement, and guiding future data collection efforts, ensuring that resources are focused on the most impactful variables.

Interestingly, even if a variable like Copper is deemed highly important globally, it might not appear in the actual decision tree structure. This discrepancy arises because decision trees make local decisions at each split point, optimizing for the best immediate split based on metrics like Gini impurity or information gain. Consequently, a variable that is highly important overall might not be the best choice for any specific split due to interactions with other variables or the presence of slightly better splits by other features. This underscores the importance of considering both global variable importance and local split decisions to fully understand and interpret the model’s behavior.

This study successfully demonstrates the application of ICP-MS combined with decision tree algorithms for the geographical discrimination of Greek “Gigantes Elefantes” beans, achieving high accuracy, sensitivity, and specificity. The use of the present analytical technique as a powerful elemental fingerprinting tool provides a reliable method for verifying product authenticity. The study of two specific geographical regions, Kastoria and Prespes, may limit the generalizability of the findings to other regions or similar crops. However, this study provides a valuable initial contribution to the elemental fingerprinting of the agricultural product “Gigantes Elefantes” beans, particularly given the absence of prior studies on this specific product. Additionally, according to our knowledge, decision tree models are utilized for the first time combined with multi-elemental analysis for the discrimination of the geographical origin of the samples, offering transparency and interpretability. Future research could explore the application of this methodology to “Gigantes Elefantes” beans which are cultivated in other areas and/or countries, expanding the scope of geographical discrimination studies.

## 4. Conclusions

In conclusion, in the present study, an assessment of the multi-elemental composition of giant beans “Gigantes Elefantes” from two cultivation areas of Greece was performed. In total, 280 samples from the two study areas (Prespes and Kastoria) and from two harvesting years (2021 and 2022) were analyzed, investigating the possibility of discriminating the geographical origin of the samples from the two tested areas. To accomplish this, the resulting elemental composition dataset of the ICP-MS analysis was processed using decision tree algorithms. The obtained model achieved high accuracy (92.86%), sensitivity (87.50%), and specificity (96.88%), suggesting an excellent tool for geographical origin discrimination, from the food traceability scope even in the case of such similar geological areas. The present work could be beneficial in future studies, which will recommend the potential use of multi-elemental profiles to discriminate the PDI “Gigantes Elefantes”, originating from Prespes and Kastoria, from unidentified imported products. 

## Figures and Tables

**Table 1:** Comparison of our results and literature data for white common beans.

Element *[mg/kg]*	KASTORIA 2021–2022	PRESPES 2021–2022	REF [20]	REF [28]
	Min–Max Values	Min–Max Values	Measured Values	Min–Max Values
**7 Li**	0–0.0297	0.0000–0.0188		
**9 Be**	0–0.0029	0.0000–0.0024		
**11 B**	16.2022–26.7011	16.6426–26.9029		
**23 Na**	0.6359–4.3197	0.4578–3.9156	9.0	1.7–38.3
**24 Mg**	2983.4903–4147.5156	2921.4996–4775.9513	1900	1400–1700
**27 Al**	0.34–5.139	0.1536–7.6771		
**31 P**	8083.4798–10,601.294	4034.3960–18,617.0031	7150	4200–5900
**39 K**	27,852.927–44,876.294	29,251.2092–49,898.9900	185,000	13,500–19,100
**44 Ca**	299.271–730.1933	340.4705–978.6075	1900	900–1300
**47 Ti**	0.5422–1.1998	0.5330–1.1649		1.6–3.8
**51 V**	0.0001–0.0026	0.0000–0.0016		0.000–0.045
**52 Cr**	0.0044–0.0272	0.0024–0.0833	0.28	0.1–0.3
**55 Mn**	24.1273–42.5852	24.9287–59.3893	15.3	9.4–13.6
**56 Fe**	62.4751–121.2127	64.2678–135.1724	86.5	56.9–77.3
**59 Co**	0.0753–1.1478	0.1176–0.9983	0.12	0.04–0.15
**60 Ni**	2.0494–21.791	0.3340–6.7266		
**63 Cu**	7.9588–18.252	7.2041	11.6	5.2–9.5
**66 Zn**	34.7405–63.3961	27.8167–57.9961	42.4	20.3–28.9
**69 Ga**	0.0655–0.6873	0.0089–0.6946		
**72 Ge**	0.0027–0.0058	0.0019–0.0059		
**75 As**	0.0007–0.0101	0.0013–0.0067		
**78 Se**	0.0078–0.3765	0.0069–0.0847		
**85 Rb**	1.3169–38.2707	2.9828–43.7599		2.4–9.0
**88 Sr**	1.5592–9.9054	1.5193–12.8573		0.8–4.6
**90 Zr**	0.0002–0.0038	0.0001–0.0067		
**93 Nb**	0–0.0018	0.0000–0.0005		
**95 Mo**	0.2807–20.4391	0.2868–8.4436	2.92	0.6–8.5
**107 Ag**	0–0.0045	0.0000–0.0657		
**111 Cd**	0.0046–0.0558	0.0042–0.0841		
**133 Cs**	0.001–0.0367	0.0005–0.0608		
**137 Ba**	0.3525–4.0112	0.0516–4.2295		
**181 Ta**	0–0.0032	0.0000–0.0007		
**182 W**	0.0007–0.0557	0.0003–0.0763		
**185 Re**	0–0.0017	0.0000–0.0003		
**205 Tl**	0–0.0021	0.0000–0.0009		
**208 Pb**	0–0.0814	0.0000–0.0198		
**238 U**	0–0.0019	0.0000–0.0071		

**Table 2:** Confusion matrix of the evaluation of the predictions of the decision tree model.

		Predicted Class	
		Prespes	Kastoria
**Actual Class**	**Prespes**	93	3
	**Kastoria**	9	63

## Data Availability

The original contributions presented in the study are included in the article/supplementary material, further inquiries can be directed to the corresponding author.

## Supplementary Materials

The following supporting information can be downloaded at https://www.mdpi.com/article/10.3390/foods13183015/s1: “Multi-Elemental Analysis and Geographical Discrimination of Greek ‘Gigantes-Elefantes’ Beans Utilizing ICP-MS and Machine Learning Models”.
