# Supplementary material for: Multi-Elemental Analysis and Geographical Discrimination of Greek “Gigantes Elefantes” Beans Utilizing Inductively Coupled Plasma Mass Spectrometry and Machine Learning Models
Source: Foods. 2024 Sep 23;13(18):3015. doi: 10.3390/foods13183015 (PMC11431413; doi:10.3390/foods13183015)
Supplement: Supplementary file 1 [file foods-13-03015-s001.zip › SI_Multi-Elemental Analysis and Geographical Discrimination of Greek 'Gigantes-Elefantes' Beans Utilizing ICP-MS and Machine Learning Models.pdf]

Supplementary Information

Table S1. Operational conditions of the ICP-MS instrument.

Parameter	Value
RF Power	1550 W
Sampling Depth	8.0 mm
Nebulizer Gas	1.05 L/min
Helium Cell Gas	4.3 mL/min
Energy Discrimination	3.0 V
S/C Temperature	2 °C

Table S2. Values of limits of detection (ppb) and quantification (ppb) and the coefficient of linearity *R* of each calibration curve.

ELEMENT	[1]LOD	[2]LOQ	[3]*R*
**^{7}Li**	0,0104	0,0316	0,9991
**^{9}Be**	0,0073	0,0221	0,9992
**^{11}B**	1,3470	4,0817	0,9991
**^{23}Na**	1,6971	5,1427	0,9991
**^{24}Mg**	0,5710	1,7304	0,9993
**^{27}Al**	0,1905	0,5774	0,9990
**^{31}P**	0,0000	0,0000	0,9994
**^{39}K**	50,6682	153,5397	0,9995
**^{44}Ca**	4,2162	12,7763	0,9991
**^{47}Ti**	0,0874	0,2650	0,9993
**^{51}V**	0,0059	0,0178	0,9989
**^{52}Cr**	0,0301	0,0911	0,9990
**^{55}Mn**	0,2064	0,6254	0,9988
**^{56}Fe**	2,1675	6,5682	0,9989
**^{59}Co**	0,0134	0,0407	0,9991
**^{60}Ni**	0,0715	0,2165	0,9987
**^{63}Cu**	0,2133	0,6462	0,9988
**^{66}Zn**	0,4834	1,4647	0,9993
**^{69}Ga**	0,0077	0,0232	0,9989
**^{72}Ge**	0,0048	0,0147	0,9994
**^{75}As**	0,0107	0,0323	0,9993
**^{78}Se**	1,6219	4,9147	0,9990
**^{85}Rb**	0,0127	0,0385	0,9989

**^{88}Sr**	0,0064	0,0195	0,999
**^{90}Zr**	0,0074	0,0224	0,9992
**^{93}Nb**	0,0049	0,0149	0,9993
**^{95}Mo**	0,0071	0,0217	0,9991
**^{107}Ag**	0,0011	0,0035	0,9990
**^{111}Cd**	0,0053	0,0160	0,9989
**^{133}Cs**	0,0034	0,0103	0,9990
**^{137}Ba**	0,0094	0,0285	0,9988
**^{181}Ta**	0,0084	0,0256	0,9993
**^{182}W**	0,0035	0,0106	0,9992
**^{185}Re**	0,0169	0,0512	0,9992
**^{205}Tl**	0,0113	0,0342	0,9991
**^{208}Pb**	0,0288	0,0871	0,9990
**^{238}U**	0,0206	0,0624	0,9991

[1]LOD: Limits of Detection; [2]LOQ: Limits of Quantitation; [3]*R*: represents the coefficient of linearity.

Table S3. Recovery rate of metals of the reference material IRMM-804 Rice Flour.

Element	Certified value (mg/kg)	Measured value (mg/kg)	%Recovery
**As**	0.049	0.045	91%
**Cd**	1.61	1.70	105%
**Cu**	2.74	3.02	110%
**Zn**	23.1	22.8	98%
**Mn**	34.2	34.9	102%
**Pb**	0.42	0.51	119%

Table S4. Elemental composition of samples from Kastoria.

**KASTORIA**								
**Element** *[mg/kg]*	**2021**				**2022**			
	**Min[1]**	**Max[2]**	**Mean[3]**	**Std[4]**	**Min[1]**	**Max[2]**	**Mean[3]**	**Std[4]**
**7 Li**	0,0000	0,0297	0,0039	0,0050	0,0008	0,0161	0,0039	0,0029
**9 Be**	0,0000	0,0029	0,0002	0,0004	0,0000	0,0025	0,0004	0,0005
**11 B**	17,1170	26,3225	20,3147	1,8274	16,2022	26,7011	19,8038	1,6927
**23 Na**	0,8313	4,3197	1,9305	0,7981	0,6359	2,1711	1,2402	0,3216
**24 Mg**	3146,5066	4147,5156	3786,9556	180,6112	2983,4903	3756,9795	3451,9194	168,5431
**27 Al**	0,4204	5,1390	1,2878	0,5417	0,3400	3,0700	0,7930	0,4084
**31 P**	8083,4798	12038,9299	9875,4785	800,9596	6838,6227	10601,9958	8305,0615	754,7907
**39 K**	27852,9271	42760,6509	36059,5392	2398,2322	34066,8734	44876,2938	38313,3568	1928,1079
**44 Ca**	299,2710	730,1933	480,1416	92,8405	304,6152	636,9422	463,8916	70,3204
**47 Ti**	0,6940	1,0373	0,8384	0,0702	0,5422	1,1998	0,6974	0,0941
**51 V**	0,0001	0,0026	0,0007	0,0005	0,0002	0,0021	0,0007	0,0003

**52 Cr**	0,0044	0,0272	0,0119	0,0054	0,0053	0,0249	0,0121	0,0045
**55 Mn**	24,1273	42,5852	32,0335	3,5430	25,9015	37,2509	29,6092	2,1340
**56 Fe**	62,4751	121,2127	90,4623	12,2599	66,8988	112,1413	82,3530	10,2241
**59 Co**	0,1538	1,1478	0,3970	0,1406	0,0753	0,7821	0,3640	0,1439
**60 Ni**	2,0494	21,7910	10,0112	4,0478	2,2026	13,5547	6,7786	2,4008
**63 Cu**	7,9588	18,2520	13,7473	1,8165	9,7651	16,0281	12,0197	1,0573
**66 Zn**	42,4568	63,3961	52,9743	5,1719	34,7405	55,7721	43,8174	4,1803
**69 Ga**	0,0739	0,4055	0,1820	0,0830	0,0655	0,6873	0,1868	0,0995
**72 Ge**	0,0031	0,0058	0,0043	0,0006	0,0027	0,0056	0,0038	0,0006
**75 As**	0,0015	0,0101	0,0034	0,0018	0,0007	0,0055	0,0026	0,0010
**78 Se**	0,0266	0,3765	0,0898	0,0726	0,0078	0,1779	0,0487	0,0328
**85 Rb**	2,9658	38,2707	12,4362	8,1952	1,3169	28,2855	10,5963	6,2113
**88 Sr**	1,5592	9,1917	5,7955	1,7519	2,0830	9,9054	5,5289	1,4479
**90 Zr**	0,0002	0,0027	0,0008	0,0004	0,0003	0,0038	0,0009	0,0004
**93 Nb**	0,0000	0,0018	0,0001	0,0002	0,0000	0,0016	0,0001	0,0002
**95 Mo**	0,8990	20,4391	3,7323	3,3746	0,2807	10,3058	3,3262	2,3975
**107 Ag**	0,0000	0,0043	0,0008	0,0008	0,0003	0,0045	0,0010	0,0008
**111 Cd**	0,0046	0,0558	0,0152	0,0061	0,0066	0,0271	0,0159	0,0047
**133 Cs**	0,0022	0,0367	0,0097	0,0088	0,0010	0,0255	0,0074	0,0064
**137 Ba**	0,3525	2,6789	1,0889	0,5146	0,3679	4,0112	1,0844	0,5710
**181 Ta**	0,0000	0,0009	0,0001	0,0002	0,0000	0,0032	0,0001	0,0004
**182 W**	0,0008	0,0557	0,0048	0,0093	0,0007	0,0174	0,0029	0,0019
**185 Re**	0,0000	0,0017	0,0001	0,0002	0,0000	0,0011	0,0000	0,0001
**205 Tl**	0,0000	0,0021	0,0002	0,0004	0,0000	0,0013	0,0001	0,0002
**208 Pb**	0,0000	0,0199	0,0053	0,0054	0,0008	0,0814	0,0123	0,0149
**238 U**	0,0000	0,0019	0,0005	0,0004	0,0000	0,0016	0,0005	0,0003

[1]Min: minimum value; [2]Max: maximum value; [3]Mean: mean value; [4]Std: standard deviation.

Table S5. Elemental composition of samples from Prespes.

**PRESPES**								
**Element**	**2021**				**2022**			
[mg/kg]	**Min[1]**	**Max[2]**	**Mean[3]**	**Std[4]**	**Min[1]**	**Max[2]**	**Mean[3]**	**Std[4]**
**7 Li**	0,0000	0,0077	0,0010	0,0013	0,0003	0,0188	0,0044	0,0027
**9 Be**	0,0000	0,0024	0,0002	0,0004	0,0000	0,0016	0,0004	0,0004
**11 B**	16,6426	26,5563	20,1995	2,0896	16,0729	26,9029	19,8565	2,1982
**23 Na**	0,4578	3,9156	1,8279	0,8391	0,4494	2,6803	1,5027	0,5166
**24 Mg**	3000,7844	4775,9513	3925,6444	284,9897	2921,4996	3614,6049	3257,3170	184,1568
**27 Al**	0,3239	7,6771	1,5092	1,1451	0,1536	2,9557	0,5812	0,5973
**31 P**	4034,3960	18617,0031	13289,6496	1556,9765	6566,8829	9160,5093	7765,8593	729,6099
**39 K**	29251,2092	49898,9900	42314,4132	4551,3957	30222,8986	38115,2449	33917,9738	1970,2656
**44 Ca**	389,9812	978,6075	606,5756	120,5115	340,4705	620,5217	500,2538	75,1419
**47 Ti**	0,6082	1,1649	0,9112	0,0810	0,5330	0,7539	0,6388	0,0620
**51 V**	0,0000	0,0016	0,0004	0,0003	0,0001	0,0013	0,0004	0,0002
**52 Cr**	0,0024	0,0833	0,0170	0,0157	0,0036	0,0298	0,0082	0,0059
**55 Mn**	24,9287	59,3893	37,2137	5,6148	26,3266	41,3359	31,2712	3,3349

**56 Fe**	70,8627	135,1724	97,8840	12,9618	64,2678	113,2348	88,6131	10,8364
**59 Co**	0,1504	0,7432	0,3504	0,1410	0,1176	0,9983	0,3606	0,1891
**60 Ni**	0,9225	6,7266	2,4479	1,1758	0,3340	5,0985	1,9563	0,9129
**63 Cu**	7,2041	14,0287	10,5885	1,6036	6,2276	11,8426	8,4349	1,0622
**66 Zn**	33,6981	57,9961	43,2024	5,7397	27,8167	46,0613	35,7504	3,9931
**69 Ga**	0,0089	0,6946	0,2256	0,1364	0,0250	0,4776	0,1957	0,1144
**72 Ge**	0,0024	0,0059	0,0042	0,0007	0,0019	0,0053	0,0039	0,0006
**75 As**	0,0020	0,0053	0,0032	0,0009	0,0013	0,0067	0,0025	0,0009
**78 Se**	0,0069	0,0847	0,0404	0,0156	0,0086	0,0775	0,0324	0,0116
**85 Rb**	4,0063	43,7599	22,6617	12,6054	2,9828	39,5105	10,0681	6,9711
**88 Sr**	1,8567	12,8573	6,2463	2,6387	1,5193	10,0651	5,0243	2,4550
**90 Zr**	0,0001	0,0020	0,0004	0,0003	0,0004	0,0067	0,0008	0,0005
**93 Nb**	0,0000	0,0003	0,0000	0,0000	0,0000	0,0005	0,0001	0,0001
**95 Mo**	0,2868	8,4436	2,6056	2,0031	0,6355	6,3997	2,1810	1,5725
**107 Ag**	0,0000	0,0657	0,0118	0,0217	0,0002	0,0010	0,0005	0,0002
**111 Cd**	0,0042	0,0836	0,0183	0,0182	0,0054	0,0841	0,0197	0,0193
**133 Cs**	0,0020	0,0608	0,0234	0,0197	0,0005	0,0374	0,0102	0,0093
**137 Ba**	0,0516	4,2295	1,2705	0,7907	0,1572	3,0901	1,2245	0,7198
**181 Ta**	0,0000	0,0004	0,0000	0,0000	0,0000	0,0007	0,0001	0,0001
**182 W**	0,0006	0,0763	0,0069	0,0112	0,0003	0,0111	0,0028	0,0019
**185 Re**	0,0000	0,0000	0,0000	0,0000	0,0000	0,0003	0,0000	0,0000
**205 Tl**	0,0000	0,0009	0,0002	0,0003	0,0000	0,0007	0,0001	0,0002
**208 Pb**	0,0000	0,0083	0,0020	0,0014	0,0000	0,0198	0,0050	0,0047
**238 U**	0,0000	0,0071	0,0004	0,0014	0,0000	0,0006	0,0002	0,0002

[1]Min: minimum value; [2]Max: maximum value; [3]Mean: mean value; [4]Std: standard deviation.

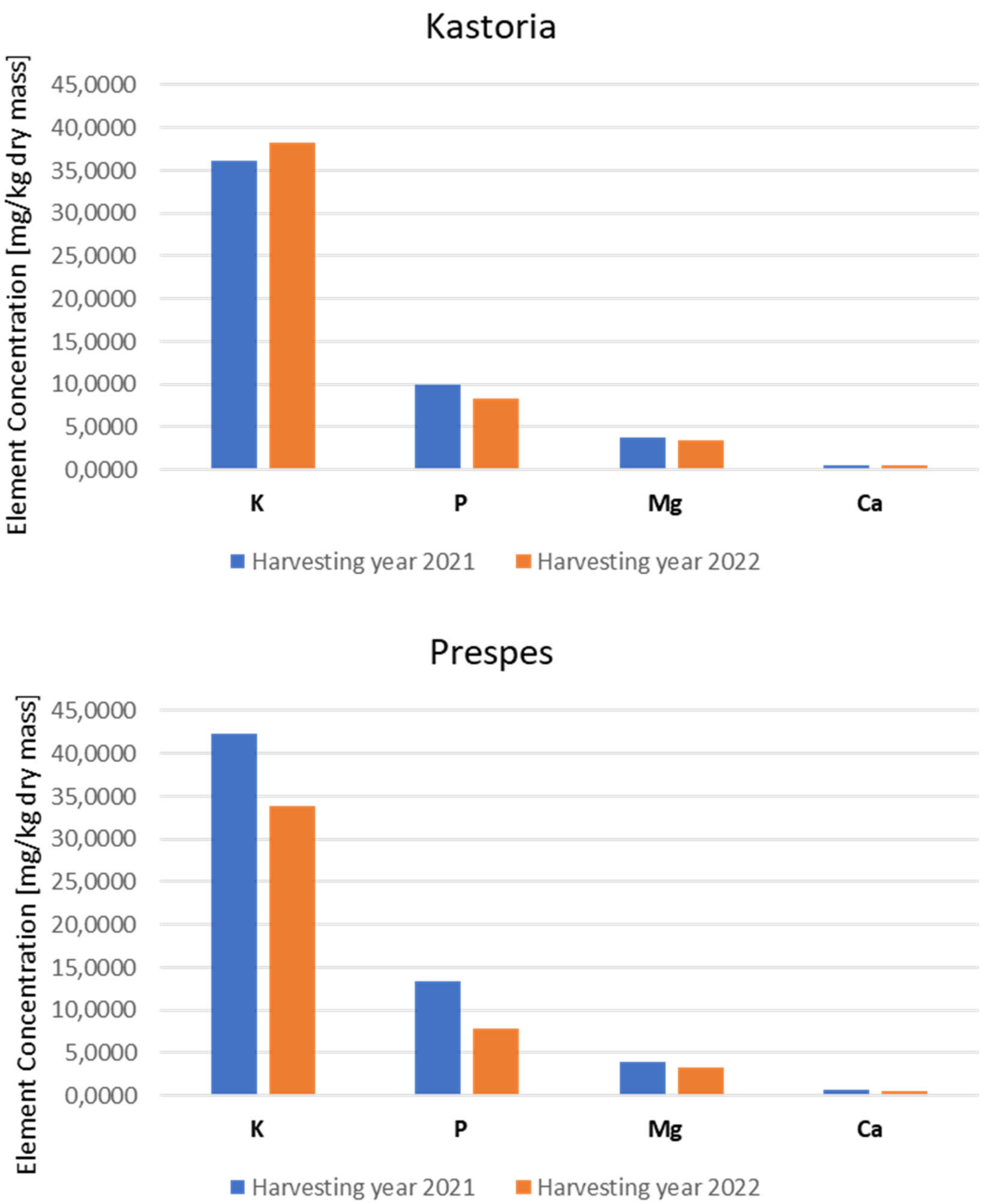

Figure S1. Comparison of the concentrations of the elements K, P, Mg, and Ca in samples from Kastoria and Prespes for the two harvesting years.

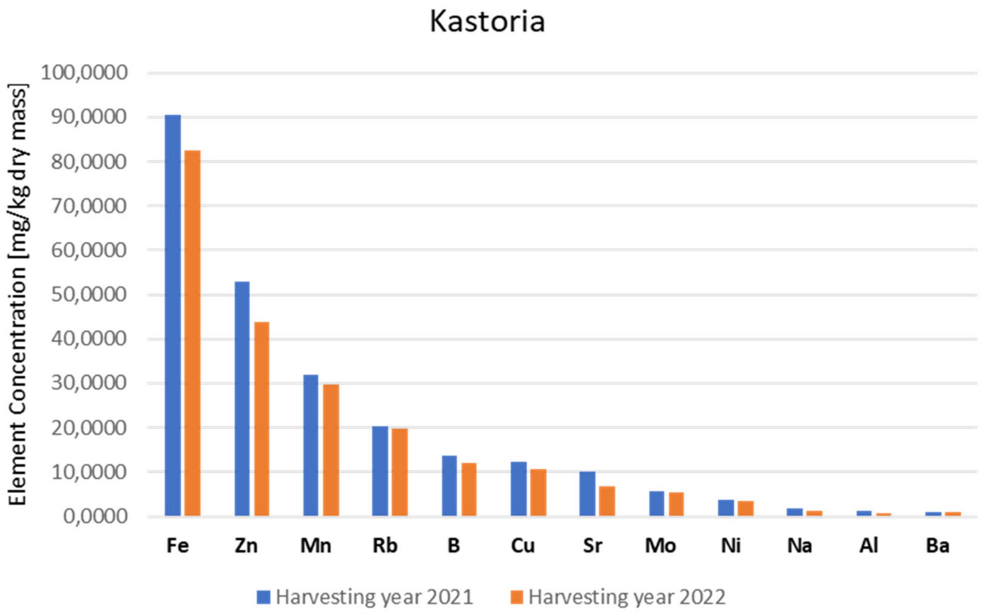

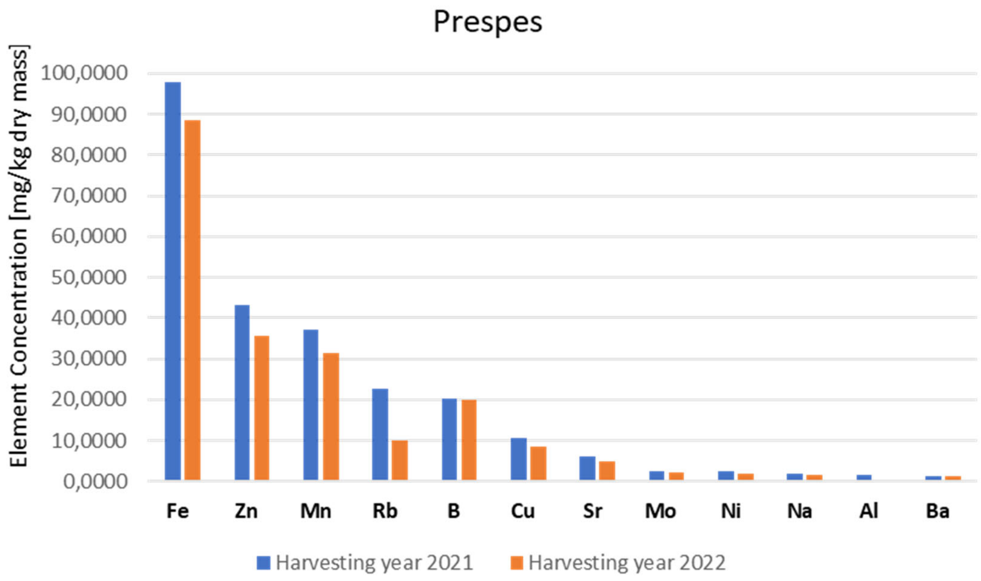

Figure S2. Comparison of the concentrations of the elements Fe, Zn, Mn, Rb, B, Cu, Sr, Mo, Ni, Na, Al and Ba in samples from Kastoria and Prespes for the two harvesting years.

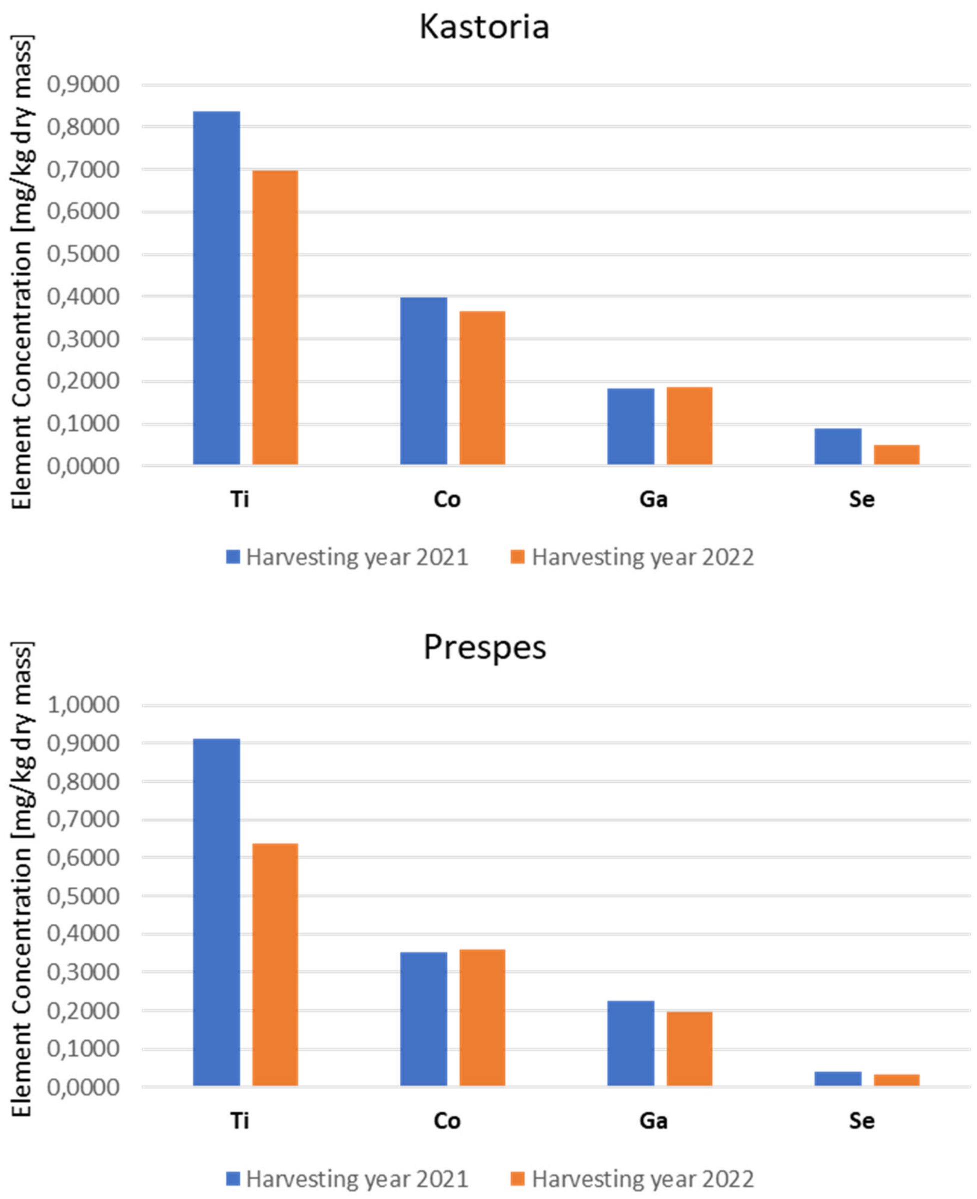

Figure S3. Comparison of the concentrations of the elements Ti, Co, Ga, and Se in samples from Kastoria and Prespes for the two harvesting years.

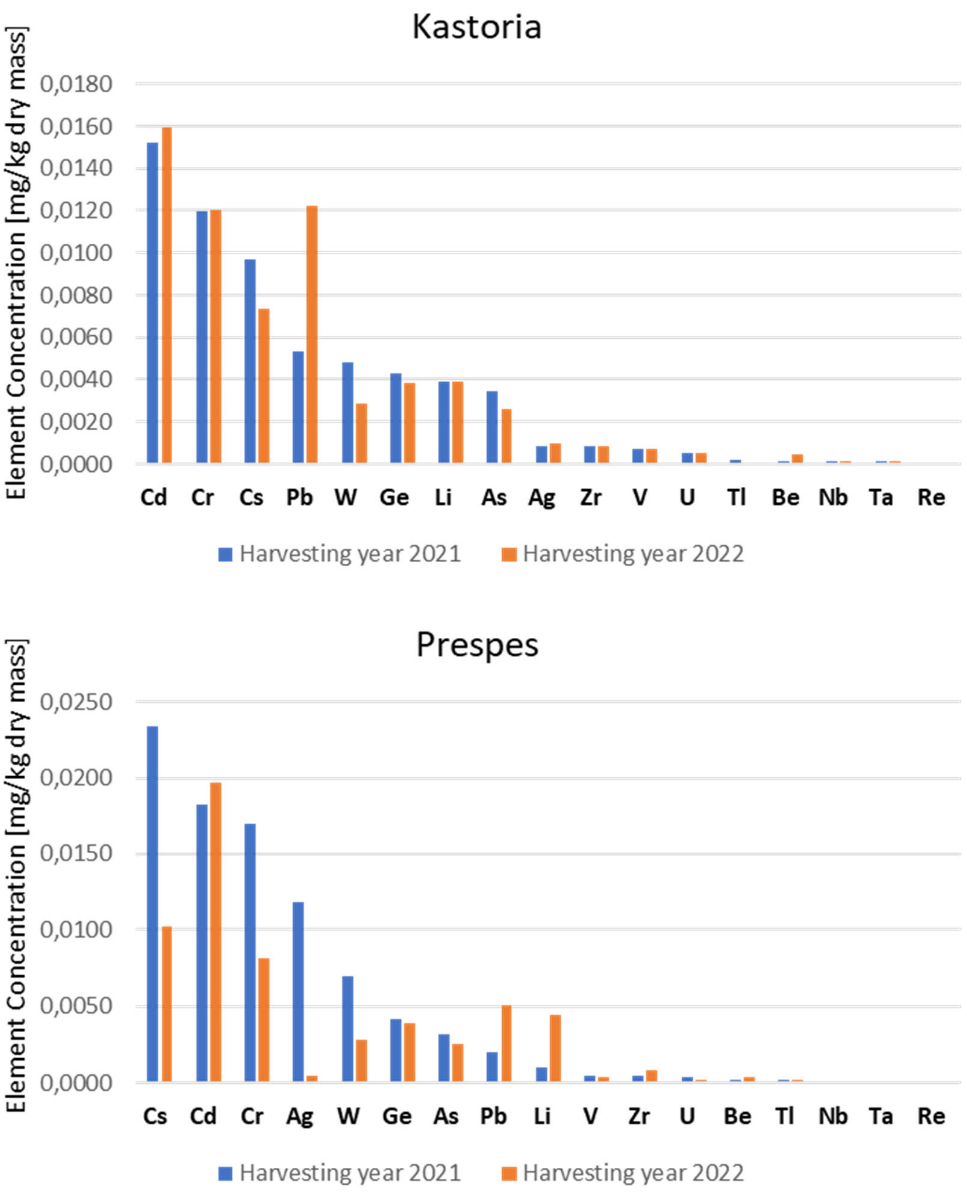

Figure S4. Comparison of the concentrations of the elements Cs, Cd, Cr, Ag, W, Ge, As, Pb, Li, V, Zr, U, Be, Tl, Nb, Ta, and Re in samples from Kastoria and Prespes for the two harvesting years.
